# Iowa University

**Published:** 1864-04

**Authors:** 


Iowa University.—The number of students attending lectures the past session was 107, and at its close the degree of M. D. was conferred on 39 candidates.

